# Probiotics Alleviate Oxidative Stress in H_2_O_2_-Exposed Hepatocytes and *t*-BHP-Induced C57BL/6 Mice

**DOI:** 10.3390/microorganisms10020234

**Published:** 2022-01-21

**Authors:** Ji Yeon Lee, Chang-Ho Kang

**Affiliations:** MEDIOGEN, Co., Ltd., Biovalley 1-ro, Jecheon-si 27159, Korea; ljy341@naver.com

**Keywords:** antioxidant, lactic acid bacteria, probiotics, HepG2 cells, *t*-BHP

## Abstract

Antioxidants protect against oxidative stress that can damage proteins, the cellular immune system, and DNA. In recent studies, probiotics have been shown to impart a microbial balance to the gastrointestinal tract, demonstrating significant antioxidant capacity. In this study, the probiotic properties and antioxidant mechanism of probiotics were evaluated in HepG2 cells and in an animal model. The characteristics of *Lactococcus lactis* MG5125, *Bifidobacterium bifidum* MG731, and *Bifidobacterium animalis subsp. lactis* MG741, which were used as lactic acid bacteria in this study, were analyzed. The results revealed the safety and stability of these probiotics in the gastrointestinal tract because they did not cause hemolysis and had excellent intestinal adhesion (75–84%). In HepG2 cells, the three probiotics alleviated H_2_O_2_-induced oxidative stress by mediating lipid peroxidation and glutathione levels and upregulating antioxidant enzymes, including catalase, superoxide dismutase, and glutathione peroxidase. In the tBHP-induced mouse model, administration of the three probiotics reduced hepatic aspartate transaminase, alanine transaminase, and lipid peroxidation levels. In conclusion, *Lc. lactis* MG5125, *B. bifidum* MG731, and *B. lactis* MG741 showed considerable antioxidant activity both in vitro and in vivo.

## 1. Introduction

Oxidative stress attacks living tissues and damages cells. In particular, reactive oxygen species (ROS), including superoxide anion (O_2_^−^), hydroxyl radical, and hydrogen peroxide (H_2_O_2_), contain oxygen with strong oxidizing power [1]. The most important organ for the oxidative energy system is the liver, which is responsible for most metabolic processes occurring in the human body [2]. Mitochondria are organelles present in hepatocytes; they decompose the consumed nutrients and generate constituent units of the substances needed by living organisms and the energy required for metabolism [3]. The electron transport system present in the mitochondrial inner membrane is a major site of ROS production; oxidative stress causes the overexpression of ROS that bind to unsaturated fatty acids in the cell membrane, causing oxidative damage to hepatocytes and inducing lipid peroxidation [2]. Therefore, a large amount of ROS leads to the breakdown of the antioxidant system, which can promote aging and cause a variety of diseases [4]. The human body has an endogenous defense system that includes Cu/Zn superoxide dismutase (SOD1), Mn superoxide dismutase (SOD2), catalase (CAT), and glutathione peroxidase (GPx), which protect cells from active oxygen by converting O_2_ to H_2_O [5]. However, the antioxidant enzymes present in the body are not sufficient to prevent oxidative stress-induced damage [6].

Probiotics, especially lactic acid bacteria, are live microorganisms that balance the microflora in the gut when administered in appropriate amounts [7]. Among them, probiotics belonging to the genera *Lactobacillus* and *Bifidobacterium* have various biological effects [8]. It is well known that the basic health-promoting function of probiotics is to reduce intestinal-related diseases by regulating and improving the intestinal microbial balance and strengthening the intestinal wall in humans [9]. Recently, probiotics have been found to benefit animal health in addition to human health [10]. Probiotics not only have beneficial effects in the colon but also have various bioactive effects, such as the alleviation of hypersensitive immune responses, the prevention of liver disease, and antioxidant and other effects [11,12,13]. In particular, the antioxidant mechanisms of *Lactococcus lactis* include the scavenging of oxidant compounds, reducing activity, chelation of metal ions, and prevention of intestinal ROS formation [6,14]. *Bifidobacterium bifidum*, which produces exopolysaccharides, exerts antioxidant activity by reducing oxidant radicals [15]. In addition, *Bifidobacterium lactis* has been reported to exhibit antioxidant effects by producing metabolites such as folic acid, glutathione (GSH), and butyrate [6].

Therefore, in this study, we investigated the probiotic properties of *L. lactis* MG5125, *B. bifidum* MG731, and *B. lactis* MG741 and assessed their antioxidant effects by evaluating toxicity, lipid peroxidation, and antioxidant enzymes (CAT, SOD, and GPx) in hepatocytes and liver tissues with induced oxidative stress.

## 2. Materials and Methods

### 2.1. Chemicals

Antibiotic strips were purchased from BioMérieux (Marcy-l’Étoile, Lyon, France). Palcam agar was purchased from Oxoid (CM0877; Hampshire, UK). Sheep blood was obtained from MBCell (Seoul, Korea). De Man, Rogosa, and Sharpe (MRS) agar and broth were purchased from Difco (Detroit, MN, USA). HepG2 and HT-29 cells were obtained from the Korea Cell Line Bank (Seoul, Korea). Minimum essential medium (MEM), Dulbecco’s modified Eagle’s medium (DMEM), fetal bovine serum (FBS), and penicillin–streptomycin (PS) were purchased from Gibco (Grand Island, NY, USA). Thiobarbituric-acid-reactive substance (TBARS) and GSH assay kits were purchased from Cayman (Ann Arbor, MI, USA). Mouse malondialdehyde (MDA) ELISA kit was obtained from LSBio (Seattle, DC, USA). GSH detection assay kit and CAT, SOD, and GPx assay kits were purchased from Abcam (Cambridge, UK). NuceloZOL for mRNA extraction was obtained from Macherey-Nagel GmbH & Co. (Dueren, Germany). The reverse transcriptase premix was purchased from Intron (Seongnam-si, Korea). The iQ™ SYBR^®^ Green Supermix was purchased from Bio-Rad (Hercules, CA, USA). All other chemicals were purchased from Sigma-Aldrich (St. Louis, MO, USA).

### 2.2. Bacterial Strain Culture and Sample Preparation

The probiotic strains (*Lc. lactis* MG5125, *B. bifidum* MG731, and *B. lactis* MG741) were provided by MEDIOGEN Co., Ltd. (Jechon, Korea). The strains were identified by 16S rRNA gene sequencing using universal rRNA gene primers (27F and 1492R) (SolGent Co., Ltd., Daejeon, Korea). Each strain was cultured in MRS broth at 37 °C in an anaerobic chamber.

Cell-free supernatant (CFS) was prepared following the procedure described by Escamilla et al. [16]. The probiotic strains grown in MRS broth were diluted to an optical density of 0.9–1.0 (10^8^–10^9^ CFU/mL) at 600 nm and then inoculated at 2% (*v*/*v*) in fresh MRS broth. After 24 h, the probiotic strains were centrifuged at 4000× *g* at 4 °C for 10 min. The supernatants were filtered through a 0.2 μm polytetrafluoroethylene membrane syringe filter (Advantec, Tokyo, Japan) and stored at −80 °C until used for the in vitro study.

For the animal study, the harvested probiotic bacterial pellets were freeze-dried. The powdered cells were harvested, mixed with maltodextrin for dilution to a cell density of 5 × 10^10^ CFU/g, and stored at 4 °C until further use.

### 2.3. Cell Culture

HepG2 cells, frequently used to model hepatocytes, were cultured in MEM; HT-29 cells, intestinal epithelial cells, were cultured in DMEM at 37 °C in a 5% CO_2_ incubator. All media used for cell culture contained 10% FBS and 1% PS. The cells were subcultured until 70–80% confluent.

### 2.4. Probiotic Properties

#### 2.4.1. Hemolytic Activity

*Lc. lactis* MG5125, *B. bifidum* MG731, and *B. lactis* MG741 were streaked on CM0877 palcam agar plates containing 5% sheep blood and incubated for 48 h at 37 °C. Hemolytic activity was determined as α (partial hemolysis), β (toxin), or γ (no hemolysis) [17].

#### 2.4.2. Antimicrobial Susceptibility Test

An antimicrobial susceptibility test was performed using antibiotic minimum inhibitory concentration (MIC) strips. The bacteria were grown for 24 h at 37 °C in MRS agar. The colonies were harvested and resuspended in phosphate-buffered saline (PBS) to 0.5 McFarland turbidity. The suspensions were smeared on brain heart infusion agar using a cotton swab. The MIC test strips were placed on the agar surface according to the manufacturer’s instructions. The plates were incubated at 37 °C, and the results were assessed after 24 h according to the European Food Safety Authority (EFSA) (Parma, Italy) guidelines [18].

#### 2.4.3. Adhesion Assay on Intestinal Epithelial Cells

Adhesion of probiotics to HT-29, the colonic epithelial cell line, was performed as described by Chen et al. with slight modifications [19]. Briefly, HT-29 cells were seeded at a density of 4 × 10^5^ cells/mL in 12-well plates under 5% CO_2_ at 37 °C until a monolayer was formed. *Lc. lactis* MG5125, *B. bifidum* MG731, and *B. lactis* MG741 (each 1 × 10^8^ CFU/mL) in DMEM without FBS and PS were added to each well and incubated for 2 h. HT-29 cells were washed with PBS to remove the nonadherent bacterial cells. The adhesion ratio (%) was calculated by comparing the number of adherent cells with the initial number of viable cells determined by plate counting.

### 2.5. Cytotoxicity Assay

Cytotoxicity was measured using the 3-(4,5-dimethylthiazol-2-yl)-2,5-diphenyltetrazolium bromide (MTT) assay [20]. Briefly, HepG2 cells were seeded at 4 × 10^5^ cell/mL in a 96-well plate and pretreated with the CFS of probiotics (2%) for 24 h followed by treatment with H_2_O_2_ (1 mM) for an additional 24 h. The hepatocytes were treated with the MTT solution (0.2 mg/mL) and incubated for 2–4 h at 37 °C. The formazan product produced by MTT was dissolved in dimethyl sulfoxide (150 μL), and absorbance was measured at 550 nm using a microplate reader (EPOCH2, BioTek, Winooski, VT, USA).

### 2.6. Measurement of Lipid Peroxidation and GSH

Lipid peroxidation based on the TBARS assay and GSH were determined according to the manufacturer’s instructions [11]. Briefly, after probiotic treatment, the cells were washed with PBS, resuspended in assay buffer, and sonicated. Lipid peroxidation, determined by MDA content, was estimated using the whole lysate, and absorbance was measured at 540 nm using a microplate reader. For GSH, cell lysate was centrifuged at 2000× *g* for 10 min at 4 °C; the supernatant was collected, and absorbance was measured at 405 nm using a microplate reader (EPOCH2, BioTek).

### 2.7. Quantitative Real-Time Polymerase Chain Reaction (qRT-PCR)

Total RNA was isolated from hepatocytes using NuceloZOL, and mRNA (1 μg) was reverse-transcribed to cDNA using reverse transcriptase premix. qRT-PCR was performed using CFX Connect Real-Time PCR Detection System (Bio-Rad) and iQ™ SYBR^®^ Green Supermix along with primers. The following primer sequences were used: CAT forward, 5-AACTGTCCCTACCGTGCTCGA-3 and reverse, 5-CCAGAATATTGGATGCTGTGCTCCAGG-3; SOD forward, 5-AATGGACCAGTGAAGGTGTGGGG-3 and reverse, 5-CACATTGCCCAAGTCTCCAACATGC-3; GPx forward, 5- CGGCCCAGTCGGTGTATGC-3 and reverse, 5-CGTGGTGCCTCAGAGGGAC-3; and glyceraldehyde-3-phosphate dehydrogenase (GAPDH) forward, 5-ACCCACTCCTCCACCTTTG-3 and reverse, 5-CTCTTGTGCTCTTGCTGGG-3′. Relative gene expression was normalized to that of GAPDH. Expression levels were analyzed using the 2^−ΔΔCT^ method [21].

### 2.8. Animal Treatment

C57BL/6 female mice (5-weeks old) were purchased from Orient Bio Inc. (Gyeonggi-do, Korea) and housed under a 12/12 h light–dark cycle (7 a.m. to 7 p.m.) in a room under controlled conditions (22 ± 3 °C, 50 ± 20% relative humidity, 150–300 Lux). The mice were fed a commercial laboratory diet (D11112201; Research Diets Inc., New Brunswick, NJ, USA) and water ad libitum. All animal experimental protocols used in this study were approved (Approval No. P214054) by the Institutional Animal Care and Use Committee at the NDIC, Gyeonggi-do, Korea.

The treatments have been described previously [22]. All animals were randomly divided into five groups (*n* = 7 in each group) and orally administered (p.o.): (1) saline (normal control, NOR), (2) *tert*-butyl hydroperoxide (*t*-BHP), (3) *Lc. lactis* MG5125 (1 × 10^9^ CFU/g/day in saline), (4) *B. bifidum* MG731 (1 × 10^9^ CFU/g/day in saline), and (5) *B. lactis* MG741 (1 × 10^9^ CFU/g/day in saline). On day 15, all animals except the NOR group were intraperitoneally (i.p.) injected with *t*-BHP (0.5 mmol/kg body weight), and the mice were sacrificed after 24 h. Blood was collected to obtain serum samples and stored at 80 °C until further experiments.

### 2.9. Biochemical Parameters

Serum samples were analyzed for alanine aminotransferase (ALT) and aspartate aminotransferase (AST) levels using a chemistry analyzer (AU480; Beckman Coulter, CA, USA). To measure MDA, GSH, CAT, SOD, and GPx activity, the liver tissues were lysed using TissueLyser II (Qiagen, Hilden, Germany), and their levels were evaluated according to the manufacturer’s instructions. Absorbance was measured using a microplate reader (SpectraMax M2; Molecular Devices, San Jose, CA, USA).

### 2.10. Statistical Analysis

Data are expressed as the mean ± standard error of the mean (SEM). Data were analyzed by multiple comparison test using one-way analysis of variance (ANOVA) followed by Duncan’s multiple range test using the IBM SPSS Statistics 21 software program (SPSS Inc., Chicago, IL, USA). The results were considered statistically significant at *p* values less than 0.05.

## 3. Results

### 3.1. Safety Test of Probiotics

**Figure 1:**
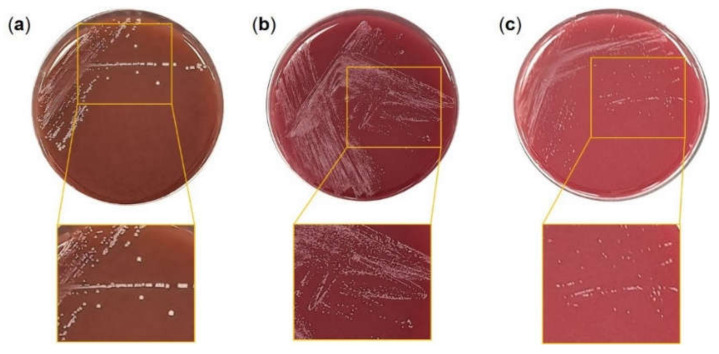
Hemolytic activity of (**a**) *Lc. lactis* MG5125, (**b**) *B. bifidum* MG731, and (**c**) *B. lactis* MG741.

*Lc. lactis* MG5125, *B. bifidum* MG731, and *B. lactis* MG741 showed white colonies with no hemolytic activity (Figure 1).

In the antimicrobial susceptibility test, *Lc. lactis* MG5125 was susceptible; however, *B. bifidum* MG731 and *B. lactis* MG741 showed resistance to gentamicin, and *B. lactis* MG741 showed resistance to streptomycin (Table 1).

### 3.2. Adhesion of Probiotics to HT-29 Cells

Probiotics must attach to intestinal epithelial cells and form colonies. Therefore, adhesion rates of *Lc. lactis* MG5125, *B. bifidum* MG731, and *B. lactis* MG741 were determined in HT-29 cells. *Lc. lactis* MG5125 (84.09 ± 0.96%), *B. bifidum* MG731 (75.37 ± 0.37%), and *B. lactis* MG741 (79.55 ± 0.32%) exhibited adhesion rates ≥75%.

### 3.3. Cytoprotective Effect of Probiotics on H_2_O_2_-Exposed Hepatocytes

**Figure 2:**
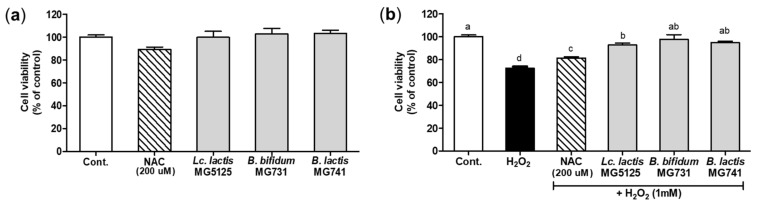
Effect of probiotics on viability of hepatocytes measured by the MTT assay. (**a**) Effect on viability of hepatocytes treated with *Lc. lactis* MG5125, *B. bifidum* MG731, and *B. lactis* MG741 (2%). (**b**) Cytoprotective effect of *Lc. lactis* MG5125, *B. bifidum* MG731, and *B. lactis* MG741 on H_2_O_2_-exposed hepatocytes. The cells were pretreated with the CFS of probiotics (2%) for 24 h and then exposed to H_2_O_2_ (1 mM) for 24 h. The data represent the mean ± SEM (*n* = 3). Different letters among columns indicate significance at *p* < 0.05, as determined by Duncan’s test. Cont., control; NAC, *N*-acetylcysteine.

The viability of hepatocytes treated with probiotics was investigated using the MTT assay (Figure 2a). A cytotoxic effect on hepatocytes was not observed after incubation with the CFS of probiotics (2%) or N-acetylcysteine (NAC), a positive control, for 24 h. The cytoprotective effect on hepatocytes treated with H_2_O_2_ (1 mM) after pretreatment with the CFS of probiotics is shown in Figure 2b. A reduction in viability was observed in cells treated with H_2_O_2_ (1 mM). In cells treated with the CFS of probiotics and H_2_O_2_, viability was significantly increased by 1.26–1.40-fold compared to H_2_O_2_ treatment (*p* < 0.05). However, all CFSs of probiotics showed a lower protective effect than that of NAC. Based on these results, 2% CFS of probiotics, which exerted a cytoprotective effect, was chosen for subsequent experiments.

### 3.4. Probiotics Elevated Antioxidant Activity in H_2_O_2_-Exposed Hepatocytes

**Figure 3:**
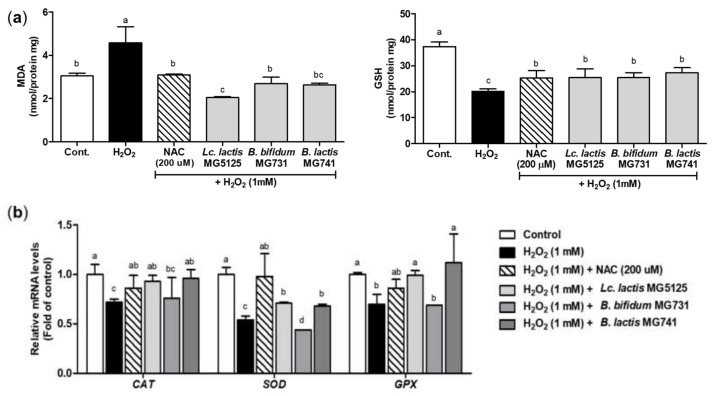
Antioxidant effect of the CFS of probiotics against oxidative stress induced by H_2_O_2_ in hepatocytes. (**a**) MDA and GSH levels in H_2_O_2_-exposed hepatocytes pretreated with the CFS of probiotics. (**b**) mRNA levels of antioxidant enzymes (CAT, SOD, and GPx) in H_2_O_2_-exposed hepatocytes pretreated with the CFS of probiotics. The cells were pretreated with the CFS of probiotics for 24 h and then exposed to H_2_O_2_ (1 mM) for 24 h. Each mRNA level was normalized to the *GAPDH*. The data represent the mean ± SEM (*n* = 3). Different letters among columns indicate significance at *p* < 0.05, as determined by Duncan’s test. Cont., control; NAC, *N*-acetylcysteine.

The MDA and GSH levels were measured to assess antioxidant activity after treating H_2_O_2_-exposed hepatocytes with the CFS of probiotics (Figure 3a). The H_2_O_2_-exposed control group showed the highest MDA level (4.87 ± 0.36 nmol/protein mg, *p* < 0.05). Conversely, in H_2_O_2_-exposed hepatocytes treated with *Lc. lactis* MG5125, *B. bifidum* MG731, and *B. lactis* MG741, the MDA level was similar or significantly lower (2.07–2.72 nmol/protein mg) than in those treated with NAC, a positive control (3.10 ± 0.11 nmol/protein mg). Moreover, compared with the control, the GSH level was reduced in hepatocytes after exposure to H_2_O_2_ (20.15 ± 0.45 nmol/protein mg). H_2_O_2_-exposed hepatocytes treated with probiotics (*Lc. lactis* MG5125, *B. bifidum* MG731, and *B. lactis* MG741) showed increased GSH levels of 25.30–27.29 nmol/protein mg (*p* < 0.05), similar to that of the positive control (25.41 ± 0.95 nmol/protein mg, *p* < 0.05).

To confirm the antioxidant activity of the CFS of probiotics in H_2_O_2_-exposed hepatocytes, the expression levels of CAT, SOD, and GPx were measured using qRT-PCR (Figure 3b). CAT, SOD, and GPx mRNA levels in cells treated with H_2_O_2_ were significantly lower than those in the control (*p* < 0.05). In H_2_O_2_-exposed hepatocytes, CAT, SOD, and GPx mRNA levels were increased after treatment with the CFS of *Lc. lactis* MG5125 (1.30-, 1.31-, and 1.41-fold higher than the H_2_O_2_ control, respectively, *p* < 0.05) and *B. lactis* MG741 (1.30-, 1.25-, and 1.60-fold higher than the H_2_O_2_ control, respectively, *p* < 0.05). However, the CFS of *B. bifidum* MG731 had no effect on antioxidant enzyme activity. These results suggest that probiotics protect hepatocytes from oxidative stress by upregulating the levels of antioxidant enzymes.

### 3.5. Probiotics Reduced Hepatic Injury in t-BHP-Induced Mice

**Figure 4:**
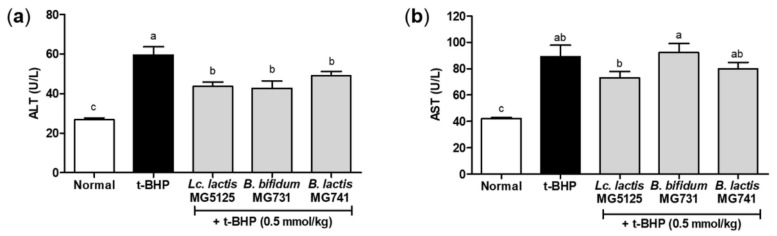
(**a**) ALT and (**b**) AST levels in the serum of *t*-BHP-induced C57BL/6 mice pretreated with probiotics for 14 days. The data represent the mean ± SEM (*n* = 7). Different letters among columns indicate significance at *p* < 0.05, as determined by Duncan’s test.

To evaluate the hepatic damage caused by oxidative stress, ALT and AST levels were measured in the serum of mice administered probiotics and *t*-BHP (Figure 4). Due to oxidative stress induced by *t*-BHP in mice, there was a significant increase in ALT and AST levels (59.57 and 89.29 U/L), but only a similar or significant increase was observed in the probiotic administration groups (42.71–49.14 and 73.14–92.14 U/L). *Lc. lactis* MG5125 and *B. lactis* MG741 administration markedly alleviated hepatic injury by reducing ALT and AST levels in the serum of *t*-BHP-induced mice.

### 3.6. Probiotics Increased Antioxidant Activity in the Liver Tissues of t-BHP-Induced Mice

The *t*-BHP-injected animal model is mainly used to observe the antioxidant effects of many compounds and extracts. To investigate the antioxidant effect of the three probiotics, MDA and GSH levels were evaluated (Table 2). Probiotics (*Lc. lactis* MG5125, *B. bifidum* MG731, and *B. lactis* MG741) suppressed the MDA level (0.61–0.68-fold of the *t*-BHP-injected group), which was similar to that in the normal group (0.66-fold of the *t*-BHP-injected group). The GSH level of *t*-BHP-injected mice was 0.87-fold that of the normal control; however, the probiotics used in this study slightly increased the GSH level (0.88–0.91-fold of the normal control). In addition, we confirmed that CAT, SOD, and GPx were affected by the administration of *Lc. lactis* MG5125, *B. bifidum* MG731, and *B. lactis* MG741 in vitro (Table 2). The probiotics used in this study restored the CAT level, lowered by *t*-BHP injection, to the same extent as in the normal group, but no change in SOD and GPx levels was observed in any group.

## 4. Discussion

Although strains of the genera Lactobacillus and Bifidobacterium are generally considered safe based on their long-term use as probiotics in humans, it is important to conduct safety assessments on specific strains because it has not been confirmed that all members can be used as probiotics [24]. To confirm *Lc. lactis* MG5125, *B. bifidum* MG731, and *B. lactis* MG741 as probiotics, a hemolytic activity test, antibiotic susceptibility test, and adhesion assay were conducted on intestinal epithermal cells. The three strains were found to be safe, as they did not cause hemolysis, showed a ≥70% adhesion rate in HT-29 intestinal cells, and were found to be stable in the colon (Figure 1). Probiotics reduce oxidative stress either by using their own antioxidant enzymes or by producing antioxidant metabolites [6]. In this study, to confirm the oxidative stress-relieving effect of probiotics, *Lc. lactis* MG5125, *B. bifidum* MG731, and *B. lactis* MG741 were evaluated based on toxicity, lipid peroxide and glutathione levels, and antioxidant enzyme mRNA expression studies conducted in cells and animal models induced by oxidative stress.

The liver is one of the most important organs for metabolism. Overexpressed ROS bind to unsaturated fatty acids of the cell membrane to induce lipid peroxidation, which is the main mechanism of oxidative damage in hepatocytes [2]. In addition, HepG2 cells are sensitive to oxidative stress because NADPH oxidase (NOX)-generated ROS mediate N-ethylmaleimide-induced K^+^-Cl^−^ cotransport activation [25]. When H_2_O_2_ treatment increased oxidative stress in hepatocytes, it damaged cells and DNA, causing cytotoxicity [26]. Additionally, *t*-BHP induced toxicity due to oxidative stress in vivo [27]. Serum AST and ALT enzyme levels are used as indicators of toxicity, and *t*-BHP injection has been reported to increase these levels [28]. These results suggest that toxicity induces apoptosis due to oxidative stress. In this study, we confirmed that treatment with probiotics (*Lc. lactis* MG5125, *B. bifidum* MG731, and *B. lactis* MG741) has protective effects on hepatocytes and hepatic tissues by preventing oxidative stress-induced toxicity (Figure 2 and Figure 4). The antioxidant effect of *Lc. lactis* MG5125, *B. bifidum* MG731, and *B. lactis* MG741 might have resulted from short-chain fatty acid production by lactic acid bacteria [29].

To protect against oxidative stress, organelles have enzymatic and nonenzymatic activities, including glutathione, thioredoxin, vitamin C, and other metabolites, and antioxidant defense systems such as SOD, CAT, and GPx [30]. Excessive oxidative stress leads to the breakdown of the antioxidant system, which can promote aging and cause a variety of diseases [26]. In this study, *Lc. lactis* MG5125 and *B. lactis* MG741 reduced MDA levels and elevated GSH levels and SOD, CAT, and GPx enzyme activity in hepatocytes. *B. bifidum* MG731 modulated MDA and GSH levels; however, no increase in antioxidant enzyme activity was observed in hepatocytes. Oxidative stress is also mediated by other antioxidant signaling pathways [6]. It has been reported that *Lactiplantibacillus plantarum* CAI6 and *Lactiplantibacillus plantarum* SC4 showed antioxidant effects by regulating the NF-E2-related factor 2 (Nrf2)–Kelch-like ECH-associated protein 1 (Keap1) pathway in the liver [31]. *Lacticaseibacillus rhamnosus* GG exerted an antioxidant effect by mediating mitogen-activated protein kinases (MAPKs) [32]. Our study suggests that *Lc. lactis* MG5125, *B. bifidum* MG731, and *B. lactis* MG741 exert an antioxidant effect in animal studies only by reducing MDA but not by affecting SOD, CAT, and GPx enzymes (Table 2); however, further studies based on other oxidation-related metabolism components are needed. Nonetheless, the strength of our study is that the ingestion of *Lc. lactis* MG5125, *B. bifidum* MG731, and *B. lactis* MG741 is inherent to the axis of the antioxidant effect of the intestine and liver.

## 5. Conclusions

**Figure 5:**
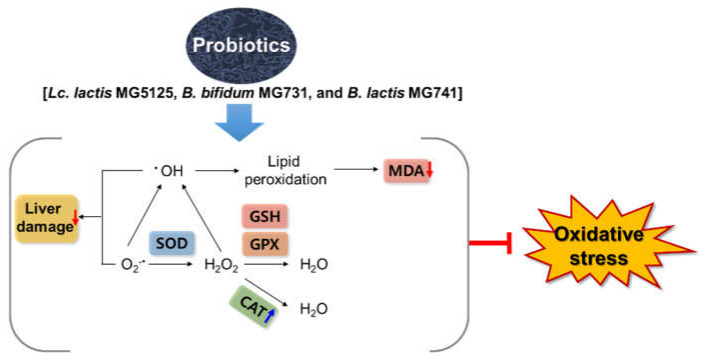
Probiotics exert a hepatoprotective effect by preventing oxidative stress in HepG2 cells and *t*-BHP-induced mice.

In conclusion, our study suggests that *Lc. lactis* MG5125, *B. bifidum* MG731, and *B. lactis* MG741 are effective in ameliorating oxidative stress in H_2_O_2_-exposed HepG2 cells and *t*-BHP-induced mice. Treatment with the three probiotics enhanced the levels of antioxidant enzymes, including SOD, CAT, GPx, and GSH, to prevent cytotoxicity in H_2_O_2_-exposed HepG2 cells. Furthermore, it was observed that administration of the three probiotics lowered serum AST and ALT levels to control toxicity and lowered the MDA level to relieve oxidative stress caused by *t*-BHP, thereby exhibiting an antioxidant effect (Figure 5). These effects suggest that changes in intestinal microflora caused by *Lc. lactis* MG5125, *B. bifidum* MG731, and *B. lactis* MG741 intake indirectly increase antioxidant effects in vivo. Therefore, the probiotics *Lc. lactis* MG5125, *B. bifidum* MG731, and *B. lactis* MG741 could serve as functional foods and therapeutic agents for preventing oxidative stress.

## Figures and Tables

**Table 1:** Determination of the MIC values of the antibiotics tested for probiotics.

Antimicrobials	*Lc. lactis* MG5125		*B.bifidum* MG731		*B. lactis* MG741	
	MIC (μg/mL)	S/R	MIC (μg/mL)	S/R	MIC (μg/mL)	S/R
Ampicillin	0.5	S	0.016	S	0.125	S
Gentamicin	2	S	>256	R	>256	R
Kanamycin	6	S	n.r	-	n.r	-
Streptomycin	32	S	48	S	384	R
Tetracycline	0.25	S	0.38	S	3	S
Chloramphenicol	16	S	0.5	S	0.75	S
Erythromycin	0.19	S	0.032	S	0.16	S
Vancomycin	0.38	S	0.5	S	0.5	S
Clindamycin	0.19	S	0.016	S	0.016	S

Susceptibility is expressed as susceptible (S) or resistant (R) according to the microbiology cut-off values from EFSA [18]. *B. lactis* MG741 showed the results reported in our previous study [23]. MIC, minimum inhibitory concentration; n.r., not required.

**Table 2:** Effect of probiotics on hepatic malondialdehyde (MDA), glutathione (GSH), catalase (CAT), superoxide dismutase (SOD), and glutathione peroxidase (GPx) levels in *t*-BHP-induced C57BL/6 mice.

Group	MDA (ng/mL)	GSH (μg/mL)	CAT (mU/mL)	SOD (%)	GPX (mU/mL)
Normal	180.19 ± 36.35 ^b^	1.28 ± 0.07	13.39 ± 1.15 ^a^	92.83 ± 0.19	61.60 ± 6.71
*t*-BHP	274.00 ± 39.77 ^a^	1.11 ± 0.06	9.71 ± 0.86 ^ab^	92.60 ± 0.29	52.03 ± 3.99
*t*-BHP + MG5125	167.60 ± 27.14 ^c^	1.13 ± 0.08	8.97 ± 1.32 ^b^	91.95 ± 0.45	47.83 ± 6.38
*t*-BHP + MG731	184.04 ± 23.67 ^b^	1.13 ± 0.10	9.40 ± 1.07 ^b^	92.47 ± 0.29	49.43 ± 7.57
*t*-BHP + MG741	185.44 ± 17.11 ^b^	1.16 ± 0.10	10.83 ± 1.69 ^ab^	92.36 ± 0.16	56.30 ± 11.47

Data represent the mean ± SEM (*n* = 7). Different letters in the same column indicate significance at *p* < 0.05, as determined by Duncan’s test.

## Data Availability

The data presented in this study are available on request from the corresponding author.
